# On the Value of Alert Systems and Gentle Rule Enforcement in Addressing Pandemics

**DOI:** 10.3389/fpsyg.2020.577743

**Published:** 2020-11-30

**Authors:** Yefim Roth, Ori Plonsky, Edith Shalev, Ido Erev

**Affiliations:** ^1^Faculty of Social Welfare and Health Sciences, Department of Human Services, University of Haifa, Haifa, Israel; ^2^Technion – Israel Institute of Technology, Haifa, Israel; ^3^The Interdisciplinary Center (IDC), Herzliya, Israel

**Keywords:** decisions from experience, rare-events, social networks, levels or reasoning, trust game

## Abstract

The COVID-19 pandemic poses a major challenge to policy makers on how to encourage compliance to social distancing and personal protection rules. This paper compares the effectiveness of two policies that aim to increase the frequency of responsible health behavior using smartphone-tracking applications. The first involves enhanced alert capabilities, which remove social externalities and protect the users from others’ reckless behavior. The second adds a rule enforcement mechanism that reduces the users’ benefit from reckless behavior. Both strategies should be effective if agents are expected-value maximizers, risk averse, and behave in accordance with cumulative prospect theory (Tversky and Kahneman, 1992) or in accordance with the Cognitive Hierarchy model (Camerer et al., 2004). A multi-player trust-game experiment was designed to compare the effectiveness of the two policies. The results reveal a substantial advantage to the enforcement application, even one with occasional misses. The enhanced-alert strategy was completely ineffective. The findings align with the small samples hypothesis, suggesting that decision makers tend to select the options that lead to the best payoff in a small sample of similar past experiences. In the current context, the tendency to rely on a small sample appears to be more consequential than other deviations from rational choice.

## Introduction

China’s success in fighting the spread of COVID-19 is attributed, at least in part, to an aggressive use of smartphone tracking applications (apps). These apps allowed authorities to identify and isolate those who might be spreading the virus (Huang et al., 2020), and punish those who violated social distancing and personal protection rules. For example, the apps issued color codes—green, yellow, or red—that indicated whether the holder poses an infection–transmission risk. A green light granted people an unrestricted pass (e.g., to the subway, work office, and other public places) and was essential for daily life. Yellow and especially red codes were extremely confining; both indicated that their holder should be quarantined and could not travel from one place to another. Identifying a person traveling with a red code was a sufficient reason to call the police. Thus, to enforce public health regulations, authorities may have severely penalized yellow or red code holders who broke quarantine.

When COVID-19 spread to western countries, their policy makers tried to emulate the success of China’s tracking apps. Yet, possibly due to privacy and civil rights concerns, authorities in many western democracies held back the development of aggressive and intrusive enforcement features. Instead, most tracking apps were designed to only alert their users, under the assumption that a reliable alert would suffice to discourage reckless behaviors. However, it is unclear whether virus-tracking apps, which only alert users and forgo regulation enforcement, are sufficiently effective in discouraging reckless behaviors.

The current research compares the effectiveness of two strategies that could guide the design of less aggressive, but potentially effective, tracking apps. One provides enhanced alerts and the other gently enforces the rules. Our comparative analysis rests on four observations. The first is that in their day-to-day life during of a pandemic, people regularly and frequently make small decisions between behaving responsibly and behaving recklessly (e.g., adhering to physical distancing guidelines or not). The second observation is that the probability that each particular decision will result in an infection is small. The third observation is that responsible behavior further decreases the chances of getting infected but often entails a small cost relative to reckless behavior, as it is more cumbersome and less convenient. The last observation is that the individual’s infection risk in a pandemic depends not only on one’s own behavior, but also on the behavior of others. Life during a pandemic presents risk even to those who maintain social distancing and other health protection guidelines. In that respect, health related behavior during pandemics is similar to driving; sharing the road with other drivers presents risk even to cautious drivers. To combat the virus, it is therefore essential to understand not only the individuals’ risk-taking behaviors but also the social dynamics that may arise in such situations. For example, it is possible that a minority of people who engage in reckless behaviors (behaviors that potentially increase the risk of infection) would make other people’s effort to behave responsibly futile, and in so doing drive otherwise responsible people to behave recklessly (Erev et al., 2020b)^1^.

Following these observations, we chose to abstract the decision environment of people in a pandemic in the context of a multi-person repeated game in which the (rare) risk imposed on each agent depends on the agent’s own decisions and the decisions of others. Specifically, we first analyzed the 4-person “Reckless or Responsible” game described in the upper panel of Table 1. This game models an environment in which reckless behavior is beneficial most of the time, but if none of the agents are reckless, behaving responsibly is the best choice on average^2^.

**TABLE 1:** Variations of the reckless or responsible game, predictions, and the observed responsible-rate.

	**Predicted responsible-rate**			
	**Cognitive hierarchy τ = 1.54**	**Naïve sampler k_*i*_ = 5**	**SAW K = 9 ω = 0.5 ε = 0.4**	**Experimental results**
**Basic setting:** Reckless: 1, 0.98; −60 otherwise (EV = −0.22) Responsible: 0 if all agents choose responsible; 0, 0.98; −60 otherwise (if at least one agent chooses Reckless)	0.11	0.09	0.18	0.09
**(2) Protecting alert app:** Reckless: 1, 0.98; −60 otherwise (EV = −0.22) Responsible: 0 with certainty	0.89	0.09	0.22	0.09
**(3.1) Always enforce app:** Reckless: [1, 0.98; −60 otherwise] – 1.2 with certainty (EV = −1.42) Responsible: 0 if all agents choose responsible; 0, 0.98; −60 otherwise (if at least one agent chooses Reckless)	0.89	1	0.87	0.85
**(3.2) Mostly enforce app:** Reckless: [1, 0.98; −60 otherwise] – [1.2, 0.95; 24 otherwise] (EV = −0.16) Responsible: 0 if all agents choose Responsible; 0, 0.98; −60 otherwise (if at least one agent chooses Reckless)	0.89	0.78	0.55	0.60

*SAW, Sampling and Weighting model (see Supplementary Appendix 1).*

The basic game has two Nash equilibria (choice profiles in which no agent wants to change choice unilaterally): An efficient equilibrium in which all agents choose “Responsible” and earn 0 with certainty, and an inefficient equilibrium whereby all agents choose “Reckless” and suffer an expected loss of 0.22. While agents should prefer the efficient equilibrium, at least two factors could impair coordination and drive them to the inefficient reckless equilibrium. The first is fear (or expectation) of reckless behavior on other agents’ side. Agents who worry that others will choose Reckless are expected to choose Reckless as well. Fear of this type is predicted, for example, by the popular “levels of reasoning” models of behavior in games (Nagel, 1995; Stahl and Wilson, 1995; Costa-Gomes et al., 2001; Camerer et al., 2004). Under such models, agents have some “level of reasoning” and play best-response to lower levels. Specifically, some agents (who are “level-0”) choose randomly and other agents (e.g., “level-1”) choose the best response to those playing level-0. Here, best-response implies acting Recklessly. As a result, higher-level agents (who chose best response to agents that are one level below them) will also choose Reckless. Furthermore, such beliefs were shown to lead to inefficient equilibria in variants of the “weakest link” game (Harrison and Hirshleifer, 1989; Van Huyck et al., 1990; Knez and Camerer, 1994), where the payoff of the individuals is affected by the lowest contributor (but see Riedl et al., 2016, for an extensive review of how to overcome such inefficiency).

A second relevant factor is that decision makers tend to select the options that lead to the best payoff in a small sample of similar past experiences (Nevo and Erev, 2012; Plonsky et al., 2015; Roth et al., 2016)^3^. In the basic game, this tendency implies a high rate of Reckless behavior because small samples are not likely to include the rare loss. For example, the probability that a 2% event will be included in a random sample of five events is only 0.096.

If agents act in line with the “level of reasoning” or “small samples” hypotheses, then most of the agents in the basic “Reckless or Responsible” game will choose to act recklessly. In search of a strategy to encourage responsible behavior we examine two variations of the basic game. The first involves elimination of the negative social externalities that lead agents to expect a higher utility from reckless behavior. This solution implies the design of an alert app that protects the agent from the reckless behavior of other agents. This would include, for example, sending alerts when approaching people who tend to exhibit reckless behavior. The second panel in Table 1 presents a variant of the basic game with a “perfectly protecting” Alert app^4^. Under this solution, the reckless behavior of others does not affect those who choose Responsible because they adhered to the alert and avoided the risk of infection. Therefore, choosing Responsible maximizes the expected value. Under the “levels of reasoning” hypothesis, the Alert app ensures that level-1 and more sophisticated agents will behave responsibly. In addition, Responsible choice minimizes risk and should be selected if losses loom larger than gains (Kahneman and Tversky, 1979) and if the agents are risk-averse (Holt and Laury, 2002) or ambiguity-averse (Fox and Tversky, 1995). Yet, under the reliance on small samples hypothesis, people choose to behave recklessly because it is better most of the time for them (regardless of the choices others make). Thus, reliance on small samples hypothesis predicts that the Alert app would have very little influence on behavior.

The second solution involves gentle rule enforcement (Erev et al., 2010; Schurr et al., 2014), i.e., a high probability that a reckless behavior will be gently penalized (without eliminating the social externalities). One way to implement gentle enforcement in a pandemic is to use tracking applications that continuously monitor a person’s behavior, and recommend the avoidance of detected reckless activities. For example, if the agent approaches a crowded place, the app will start to make an annoying sound every few seconds (similar to the seat belt beeping, see related idea in Okeke et al., 2018). The third panel in Table 1 presents a variant of the basic game that demonstrates this solution with a gentle but certain punishment (loss of 1.2 points) for each Reckless choice. Under such a regime, reckless behavior is never the best choice, and agents are expected to choose Responsible action even if they rely on small samples.

The central columns in Table 1 present the predicted “Responsible” choice rate in the current games under the “levels of reasoning” model (Cognitive Hierarchy; Camerer et al., 2004) and two abstractions of the reliance on small samples hypothesis (see Supplementary Appendix 1). The predictions of the Cognitive Hierarchy model were derived with the parameter proposed by Camerer et al. (2004). According to the basic naïve sampler model, agents would choose Responsible in the first trial, and then select the option that led to the best outcome in a random sample of five previous experiences (Erev and Roth, 2014). SAW (sampling and weighting) is a generalization of the naïve sampler model that adds noise and sensitivity to the average payoffs. The current predictions of SAW were derived with the parameters estimated in Erev et al. (2020a). Table 1 shows that the Cognitive Hierarchy model predicts that the Alert app will be as effective as the Always Enforce app, but that reliance on small samples models predicts that only the Gently Enforce app will be effective. The experiment described below was designed to test these predictions.

## Study 1A: Alert or Enforcement?

### Materials and Methods

One hundred fifty-eight MTurk workers from the USA and Canada participated in the experiment in exchange for monetary compensation. Each session included one of the three conditions: Basic (*n* = 48, 12 groups, 30 males; *M*_*age*_ = 41), Alert (*n* = 52, 13 groups, 34 males^5^; *M*_*age*_ = 34), or Enforcement (*n* = 52, 13 groups, 31 males, *M*_*age*_ = 37). Each participant could participate in only one of the sessions. The monetary payoff included a show-up fee of $1 and an additional guaranteed $2 if the participant made more than 66% of the choices (i.e., more than 40 out of the 60 choices) on time^6^ as well as a chance to earn $1 bonus, based on the number of points accumulated during the experiment^7^ (mean final pay = $3.35, see Supplementary Appendix 2 for the precise instructions).

The experiment was run in groups of four participants. Participants could proceed to the next round only after all four players made their choices. To ensure that the experiment ran smoothly, we told participants that they had 12 seconds (20 seconds the first three trials) to make their choice in each round, after which the program would automatically submit a choice for them, and they would receive a penalty of 2 points. Unbeknownst to participants, when the program auto-submitted a choice on their behalf, it made the same choice that the participant made in the previous trial (in the first trial the program auto-submitted the Responsible choice)^8^.

**FIGURE 1:**
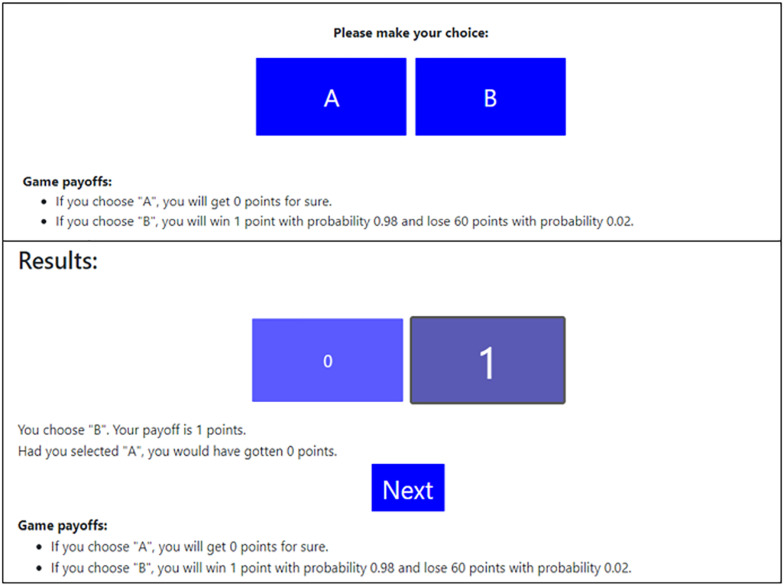
Screens presented to participants, in the “Reckless or Responsible game, “Alert” app condition. The upper image (“Please make your choice”) presents the screen at the beginning of each trial. The lower image (“Results”) presents the screen at the end of each trial.

The experiment, programmed with OTree (Chen et al., 2016), employed a variant of the clicking paradigm (Barron and Erev, 2003). In each of the 60 trials, the participants deliberated between two keys, “A” and “B.” Unbeknownst to the participants, “A” always represented the Responsible choice, while “B” represented the Reckless choice. Participants saw a complete description of the incentives structure and after each trial received feedback regarding their obtained and forgone payoff (see Figure 1).

### Results

**FIGURE 2:**
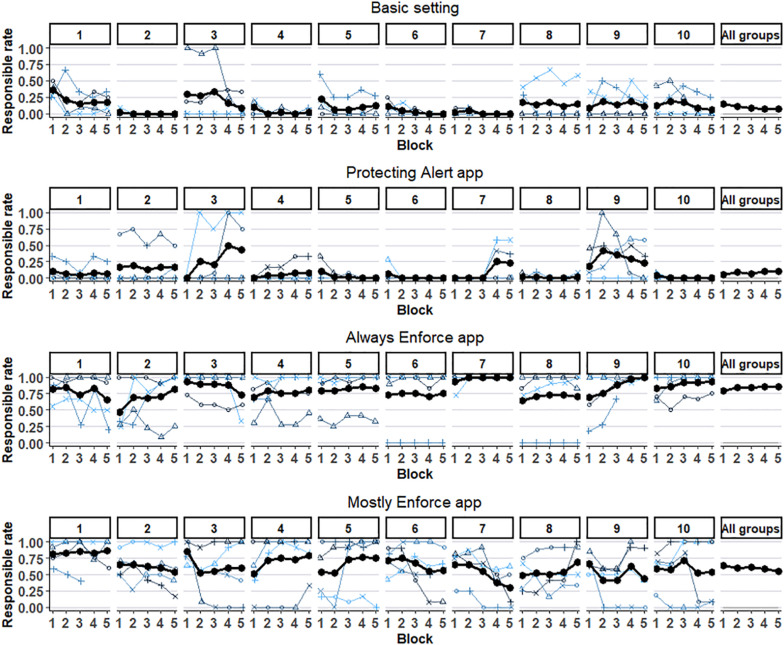
Individual and Group level “Responsible choice rates in the “Basic” (up), “Alert” (second), “Always Enforce” (third), and “Mostly Enforce” (down) conditions. Only the first 10 groups in each condition, and the mean over all groups in that condition, are shown. Each line represents a Responsible choice rate in five blocks (of 12 trials) by a participant in the respective group and condition. The bold line shows the mean Responsible choice rate of the group. The rightmost plot (of each condition) presents the overall mean Responsible rate of the respective condition.

The right-hand column in Table 1 presents the mean choice rate of the responsible option (Responsible-rate) in the first three conditions. The rates are 9% (*SD* = 7.7%), 9% (*SD* = 9.8%), and 85% (*SD* = 10.3%) in the basic, alert, and always enforce conditions, respectively. This suggests that the alert app was ineffective, while the enforcement app was highly effective in increasing the Responsible-rate. The difference between the Responsible choice rate in the basic and the alert conditions is insignificant, Welch *t*(22.5) = 0.21. The difference between the Responsible rates in the basic and the enforcement condition is significant, *t*(22.1) = −20.78, 95% CI [68.0, 83.0], and so is the difference in Responsible rates between the enforcement and alert conditions, *t*(24) = 19.3, 95% CI [68.0, 84.3]. Responsible rates are not driven by outlier groups but represent a general pattern. The responsible rates are lower than 30% in all basic and alert groups and higher than 66% in all enforcement groups. Figure 2 presents the effect of the experience on each participant in the first 10 groups, and over all groups. The recurring pattern is of relatively flat curves, with a tendency to converge toward an equilibrium.

The similar Responsible rates in the basic and the alert conditions suggest that in the current setting participants neglected the social externalities (i.e., the impact that their behavior had on others; Coase, 1960) associated with their actions. These results are consistent with previous research (Falk and Szech, 2013; Bartling et al., 2020), but more extreme (complete ignorance to the impact on others). Yet, analysis of the Responsible-rate in the very first trial reveals that the initial (pre-experience) tendency is inconsistent with complete neglect of social externalities. The initial Responsible choice rates are 28% (*SD* = 20.0%) and 15% (*SD* = 27.4%) in the Basic and Alert conditions, respectively. While the difference between these conditions is not statistically significant, *t*(22.67) = 1.67, this may just be due to lack of power. Hence, initially, participants were more likely to make responsible choices when their actions involved social externalities (Basic) than when they did not (Alert). Still, the effect appears to be small. One possible *ad hoc* explanation is shared guilt (Inderst et al., 2019), according to which people assume that even if they choose to act responsibly, others would choose to behave recklessly; therefore, the individuals’ choice to act recklessly and the guilt associated with it are attributed to others. This explanation is consistent with the fact that the effect of social externalities dissipates over time, when participants see that being responsible is pointless.

Notice that the instructions in the Alert condition imply an individual choice task. Thus, the low initial Responsible rate (only 15%) questions the generality of the tendency to overvalue rare events decisions from description; the results reveal undervaluing of rare events from description and from experience. This pattern supports the assertion that the tendency to overvalue rare events in decisions from description is not a general phenomenon; it appears to be sensitive to the framing of the choice task (see Harbaugh et al., 2010; Marchiori et al., 2015).

## Study 1B: Probability of Success, Expected Return, or Dominance?

Under the reliance on small samples hypothesis, the effectiveness of the enforcement application in Study 1a is triggered by the fact that it ensures that the payoff from responsible behavior is higher than the payoff from reckless behavior in most small samples. Study 1b was designed to compare this explanation to two alternative explanations to the effectiveness of the enforcement in Study 1a. The first is that the effect is triggered by the large decrease in the expected return from reckless behavior implied by the enforcement app. In Study 1a, enforcement decreased the expected return from reckless behavior by 1.2 points and implied a decrease of 120% from the maximal payoff. The second alternative explanation is that the effect of the enforcement in Study 1 results from the dominance of the Responsible choice; it ensured that Responsible always led to better payoff than Reckless.

In order to compare the three explanations, we designed a new condition, simulating a tracking app that does not decrease the expected return of the Reckless choice and does not make Responsible the dominant choice. Specifically, this “Mostly Enforce” app leads to a loss of 1.2 points 95% of the time and to a gain of 24 points 5% of the time. Thus, it increases the expected payoff from Reckless behavior (the expected change is −1.2(0.95) + 24^∗^(0.05) = + 0.06), and leads to better outcomes than Responsible 5% of the time. Yet, the reliance on small samples hypothesis predicts that it will enhance responsible choices relative to the basic setting. The lower panel in Table 1 presents the implied payoff distribution, and the predictions of the two quantifications of the reliance on small samples hypothesis is considered here.

The Mostly Effective app abstracts natural settings in which the effort to enforce a specific behavior increases the expected benefit from selecting it. For example, consider a service provider (e.g., a plumber or a hairdresser) who is recklessly attempting to serve as many clients as possible. In the rare cases that this attempt goes unpunished, the service provider gets increased utility since competition is scarce.

### Materials and Methods

Forty-eight^9^ MTurk workers participated in the Mostly Enforcing app game (*n* = 48, 12 groups, 29 males, *M*_*age*_ = 38) in exchange for monetary compensation. This *post hoc* study used the same procedure as the main study, but with a different payoff structure for reckless behavior (see lower panel of Table 1).

### Results

The mean Responsible rate was 59.8% (*SD* = 11.8%). This rate is significantly different from the basic [*t*(19.0) = 12.37, 95% CI [41.8, 58.9]], alert [*t*(21.6) = 11.7, 95% CI [42.0, 60.1]], and always enforcing [*t*(21.9) = 5.65, 95% CI [15.9, 34.3]] conditions. Thus, although the mostly enforce app was inferior to the always enforce app, it was still effective in increasing the Responsible choice rate compared to the basic app, and much more effective than the alert app. The latter result is rather illuminating in light of the expected value of Reckless choice (−0.22 in the alert app vs. −0.16 in the mostly enforce app conditions). In other words, on average, reckless behavior is *less* harmful in the mostly enforce app, but it is chosen more than twice *more* often in the alert app (40 vs. 92%). Furthermore, if the participants believe that at least one other participant will choose Reckless, this is the EV maximizing alternative (which is not the case in the Alert app condition).

## General Discussion

Our analysis distinguishes between two contributors to reckless behaviors that can spread infection during a pandemic. The first involves the belief that the effort to behave responsibly is pointless; it cannot reduce the probability of infection because other members of the decision makers’ social network are likely to behave recklessly. Beliefs of this type are predicted, for example, based on the hierarchical levels of reasoning model of social behavior. The second contributor involves the tendency to undervalue rare events. This tendency is predicted under the reliance on small samples hypothesis. Understanding the relative importance of the two contributors can help predict the impact of different policies designed to facilitate responsible behavior. Assuming that the main contributor is the belief that other members will behave recklessly, responsible behavior can be enhanced by effective alert systems. However, if the main contributor is reliance on small samples, alert systems are not likely to be effective, and enforcement is necessary.

The current experiments compare the relative importance of the two contributors in an abstract 4-person game. The results support the prediction of the reliance on small samples hypothesis. Simulated alert applications, expected to facilitate responsible behavior under the hierarchical levels of reasoning model, had no effect. In contrast, simulated enforcement systems were found to be highly effective. In addition, our results demonstrate that enforcement can be effective even if it does not use harsh punishments and does not reduce the expected return from reckless choices. When responsible behavior implies an efficient Nash equilibrium (the environment examined here), it is enough to ensure that the enforcement increases the probability that responsible behavior leads to the best possible payoffs toward 1 (to 0.95 in the current study, see similar observation in Erev et al., 2019).

Our results should be viewed in light of the fact that the experimental studies focused on a simplified abstract setting that differs from natural pandemics-related dilemmas in many ways. For example, to avoid framing and impression management effects, our participants did not know that we aimed to study behavior in a pandemic. It is possible that some people are more (or less) prosocial in making these less abstract decision choices (Campos-Mercade et al., 2020). Furthermore, in our setting, people were fully informed about the potential consequences of their actions and their probabilities. In real life, this is unlikely, and misinformation may also be a highly relevant factor (Bursztyn et al., 2020). Also, we chose to focus on a static setting in which the outcomes and their corresponding probabilities do not change over time or as a function of the participants’ decisions, or the policies that are set forth. This is clearly a simplification of the highly dynamic nature of a pandemic. Finally, the current study compares potential policy solutions that implicitly assume universal and mandatory adoption of the suggested apps. For example, in the enforcement conditions of our experiments, one could not simply “uninstall the application” and avoid the gentle punishments associated with reckless behaviors. In most western democracies, mandatory tracking is probably unlikely. In a follow-up study (Plonsky et al., 2020), we investigated the potential of a voluntary gentle enforcement app and showed that with smart design, it can get significant traction. Despite these limitations, we believe our results can be of significant practical value as they highlight some of the basic choice tendencies people have in decision-making settings that have the same general structure we study (games with rare negative events and social externalities), like pandemics.

## Data Availability Statement

The raw data supporting the conclusions of this article will be made available by the authors, without undue reservation.

## Ethics Statement

The studies involving human participants were reviewed and approved by the University of Haifa Faculty of Social Welfare Ethical Review board. The participants provided their written informed consent to participate in this study. The patients/participants provided their written informed consent to participate in this study.

## Author Contributions

YR, OP, and IE conceptualized the ideas. YR prepared, ran and analyzed the results of the experiments. IE prepared and ran the simulation models. All authors wrote and agreed upon the final version of the manuscript.

## Conflict of Interest

The authors declare that the research was conducted in the absence of any commercial or financial relationships that could be construed as a potential conflict of interest.

## Footnotes

1 For instance, consider a cautious driver who prefers to slow down when a traffic light changes from green to orange. If the car behind does not show signs of slowing down, it is safer for the cautious driver to speed up and drive through the orange light to increase the chance of avoiding an accident.

2 More specifically, the frequent (98% of the time) “+1” from Reckless behavior represents the mild advantage of behaving recklessly (e.g., the convenience of not wearing a mask) over the somewhat inconvenient responsible behavior. The 2% chance for the large negative payoff “−60” captures a rather wide range of “highly costly” events associated with pandemic. The most obvious of these events is getting infected and turning ill. Another highly costly event involves being in contact with a person, who later tests positive for the virus, and entering quarantine. Other negative consequences may result from unintended transmission of the virus to others or being forced into a regional lockdown (each reckless person slightly increases the chances that a local lockdown would take place). While there is more than one way to model the basic game from Table 1 (e.g., one may assign different values for the payoffs), the defining feature of the game lies in its conceptual structure: Behaving recklessly is usually more rewarding as the probability of a negative event is low. Yet reckless behavior also results in lower expected value because it occasionally leads to highly costly outcomes.

3 While it is hard to know which past experiences agents consider “similar,” the small sample of similar past experiences can be approximated well by assuming that agents rely on small *random* samples of past experiences. This assumption has been shown to predict behavior well in recent choice prediction competitions (Erev et al., 2017; Plonsky et al., 2019).

4 Clearly, this is an overly optimistic assumption: It is virtually impossible to design a perfectly protecting app. Still, this assumption is useful as a concept sensibility test (if the perfectly protecting app is ineffective there is no reason to assume that a less than perfect one will, although if it will work additional investigations are necessary).

5 Four participants did not report their age and gender.

6 The instructions were that the $2 will be given only if the subject made “most of the choices” on their own.

7 For each participant, the computerized program generated, unknowingly to participants, a random threshold (from a uniform distribution between “−20” and “+20”). If the accumulated number of points of the participant succeeded the generated threshold s/he received that additional 1$ bonus (and nothing otherwise). For example, if for a certain participant the randomly generated threshold was “−5” and at the end of the experiment this participant accumulated 7 points they received a 1$ bonus (while if the total number of the accumulated points was −9 than no bonus was provided).

8 Originally there were 272 participants. The analysis focuses on the choices made by the participants when they responded within the time limit (12 s in most trials). Groups in which more than 20% of the choices were made after the time limit (and the computer repeated the last choice) were not included in the analysis at all. The reason for the high exclusion rate is coordination: often the participants have to wait for a certain amount of time before other participants enter the group. It is common that by the time the group is formed, the first participant already voluntarily dropped out the experiment. Including all groups (or using other cutoff rather than the 20%) does not meaningfully change the mean Responsible-rates.

9 Originally there were 80 participants. We implemented the same cutoff (20% auto submissions) as in study 1. Using any other cutoff does not change the main results in any meaningful way.

## References

[B1] Barron G. Erev I. (2003). Small feedback-based decisions and their limited correspondence to description-based decisions. *J. Behav. Decis. Mak.* 16 215–233. 10.1002/bdm.443

[B2] Bartling B. Fehr E. Özdemir Y. (2020). *Does Market Interaction Erode Moral Values?* Available at: SSRN: https://ssrn.com/abstract=3683775 (accessed at August 25, 2020).

[B3] Bursztyn L. Rao A. Roth C. Yanagizawa-Drott D. (2020). *Misinformation During a Pandemic*, Working Paper, (2020–2044), Chicago: University of Chicago.

[B4] Camerer C. F. Ho T. H. Chong J. K. (2004). A cognitive hierarchy model of games. *Q. J. Econ.* 119 861–898. 10.1162/0033553041502225 32495221

[B5] Campos-Mercade P. Armando M. Schneider F. Wengström E. (2020). *Prosociality Predicts Health Behaviors During the COVID-19 Pandemic*, Working Paper, No. 346, Zurich: University of Zurich 10.5167/uzh-187672. PMC7842154 33531719

[B6] Chen D. L. Schonger M. Wickens C. (2016). oTree—An open-source platform for laboratory, online, and field experiments. *J. Behav. Exp. Finance* 9 88–97. 10.1016/j.jbef.2015.12.001

[B7] Coase R. H. (1960). “The problem of social cost,” in *Classic Papers in Natural Resource Economics*, ed. Gopalakrishnan A. (London: Palgrave Macmillan), 87–137. 10.1057/9780230523210_6

[B8] Costa-Gomes M. Crawford V. P. Broseta B. (2001). Cognition and behavior in normal-form games: an experimental study. *Econometrica* 69 1193–1235. 10.1111/1468-0262.00239

[B9] Erev I. Roth A. E. (2014). Maximization, learning, and economic behavior. *Proc. Natl. Acad. Sci. U.S.A.* 111(Suppl. 3), 10818–10825. 10.1073/pnas.1402846111 25024182 PMC4113920

[B10] Erev I. Ert E. Plonsky O. Roth Y. (2020a). *Six Contradicting Deviations from Rational Choice, and the Impact of Experience*, Working Paper.

[B11] Erev I. Ert E. Plonsky O. Cohen D. Cohen O. (2017). From anomalies to forecasts: toward a descriptive model of decisions under risk, under ambiguity, and from experience. *Psychol. Rev.* 124 369–409. 10.1037/rev0000062 28277716

[B12] Erev I. Gilboa Freedman G. Roth Y. (2019). The impact of rewarding medium effort and the role of sample size. *J. Behav. Decis. Mak.* 32 507–520. 10.1002/bdm.2125

[B13] Erev I. Ingram P. Raz O. Shany D. (2010). Continuous punishment and the potential of gentle rule enforcement. *Behav. Processes* 84 366–371. 10.1016/j.beproc.2010.01.008 20096753

[B14] Erev I. Plonsky O. Roth Y. (2020b). Complacency, panic, and the value of gentle rule enforcement in addressing pandemics. *Nat. Hum. Behav.* 4 1095–1097. 10.1038/s41562-020-00939-z 32796924

[B15] Falk A. Szech N. (2013). Morals and markets. *Science* 340, 707–711. 10.1126/science.1231566 23661753

[B16] Fox C. R. Tversky A. (1995). Ambiguity aversion and comparative ignorance. *Q. J. Econ.* 110 585–603. 10.2307/2946693

[B17] Harbaugh W. T. Krause K. Vesterlund L. (2010). The fourfold pattern of risk attitudes in choice and pricing tasks. *Econ. J.* 120 595–611. 10.1111/j.1468-0297.2009.02312.x

[B18] Harrison G. W. Hirshleifer J. (1989). An experimental evaluation of weakest link/best shot models of public goods. *J. Polit. Econ.* 97 201–225. 10.1086/261598

[B19] Holt C. A. Laury S. K. (2002). Risk aversion and incentive effects. *Am. Econ. Rev.* 92 1644–1655. 10.1257/000282802762024700

[B20] Huang Y. Sun M. Sui Y. (2020). *How Digital Contact Tracing Slowed Covid-19 in East Asia. Harvard Business Review.* Available at https://hbr.org/2020/04/how-digital-contact-tracing-slowed-covid-19-in-east-asia (accessed at April 15, 2020).

[B21] Inderst R. Khalmetski K. Ockenfels A. (2019). Sharing guilt: How better access to information may backfire. *Manag. Sci.* 65, 3322–3336. 10.1287/mnsc.2018.3101 19642375

[B22] Kahneman D. Tversky A. (1979). Prospect theory: an analysis of decision under risk. *Econometrica* 47 263–292. 10.2307/1914185

[B23] Knez M. Camerer C. (1994). Creating expectational assets in the laboratory: coordination in ‘weakest-link’games. *Strateg. Manag. J.* 15 101–119. 10.1002/smj.4250150908

[B24] Marchiori D. Di Guida S. Erev I. (2015). Noisy retrieval models of over-and undersensitivity to rare events. *Decision* 2:82 10.1037/dec0000023

[B25] Nagel R. (1995). Unraveling in guessing games: an experimental study. *Am. Econ. Rev.* 85 1313–1326.

[B26] Nevo I. Erev I. (2012). On surprise, change, and the effect of recent outcomes. *Front. Psychol.* 3:24. 10.3389/fpsyg.2012.00024 PMC3283116 22363303

[B27] Okeke F. Sobolev M. Dell N. Estrin D. (2018). “Good vibrations: can a digital nudge reduce digital overload?,” in *Proceedings of the 20th International Conference on Human-Computer Interaction with Mobile Devices and Services*, (Spain: Barcelona), 1–12.

[B28] Plonsky O. Apel R. Ert E. Tennenholtz M. Bourgin D. Peterson J. C. (2019). Predicting human decisions with behavioral theories and machine learning. *arXiv* [preprint]. Available at: https://arxiv.org/abs/1904.06866 (accessed November 18, 2020). 10.1038/s41562-025-02267-6 40691307

[B29] Plonsky O. Roth Y. Erev I. (2020). *Underweighting of Rare Events in Social Interactions and its Implications to the Design of Voluntary Health Applications.* *arXiv [preprint].* Available online at: https://psyarxiv.com/9q3db (accessed November 18, 2020).

[B30] Plonsky O. Teodorescu K. Erev I. (2015). Reliance on small samples, the wavy recency effect, and similarity-based learning. *Psychol. Rev.* 122 621–647. 10.1037/a0039413 26075914

[B31] Riedl A. Rohde I. M. T. Strobel M. (2016). Efficient coordination in weakest-link games. *Rev. Econ. Stud.* 83 737–767. 10.1093/restud/rdv040

[B32] Roth Y. Wänke M. Erev I. (2016). Click or skip: the role of experience in easy-click checking decisions. *J. Consum. Res.* 43 583–597. 10.1093/jcr/ucw053

[B33] Schurr A. Rodensky D. Erev I. (2014). The effect of unpleasant experiences on evaluation and behavior. *J. Econ. Behav. Organ.* 106 1–9. 10.1016/j.jebo.2014.05.012

[B34] Stahl D. O. Wilson P. W. (1995). On players’ models of other players: theory and experimental evidence. *Games Econ. Behav.* 10 218–254. 10.1006/game.1995.1031

[B35] Tversky A. Kahneman D. (1992). Advances in prospect theory: cumulative representation of uncertainty. *J. Risk Uncertain.* 5 297–323. 10.1007/bf00122574

[B36] Van Huyck J. B. Battalio R. C. Beil R. O. (1990). Tacit coordination games, strategic uncertainty, and coordination failure. *Am. Econ. Rev.* 80 234–248.

