# Supplementary material for: On the Value of Alert Systems and Gentle Rule Enforcement in Addressing Pandemics
Source: Front Psychol. 2020 Nov 30;11:577743. doi: 10.3389/fpsyg.2020.577743 (PMC7733921; doi:10.3389/fpsyg.2020.577743)
Supplement: Supplementary file 1 [file Data_Sheet_1.pdf]

Appendix 1 – Learning Models

The Cognitive Hierarchy model (CH, Camerer, Ho & Chong, 2004)

CH assumes that each agent is of one of k types. Agent of type Step-0 (or level-0) randomize; and Step-*k* thinkers best-respond, assuming that other players are distributed over Step-0 through Step-*k* -1 categories. The shape of the assumed distribution of type is Poisson. Camerer et al. estimated the single parameter of the Poisson distribution in large number of games and suggest that "assuming τ value of 1.5 could give reliable predictions for many other games as well." This value implies that 22% of the agents are Step-0.

The Naïve Sampler model (Barron & Erev, 2003)

The model assumes random choice in the first trial ($t = 1$), and sampling-based decisions in subsequent trials. The decision at $t > 1$ starts by randomly sampling κ previous trials with replacement (κ, a positive integer, is a free parameter). Then, the option with the largest average payoff in the sample is selected. Following Erev and Roth (2014) the current implementation assumes $\kappa = 5$.

The Sampling and Weighting (SAW) model (Erev et al., 2020)

SAW generalizes the Noisy naïve model. It assumes two response modes: exploration that implies random choice and exploitation that implies reliance on samples of 5 past experience (like the naïve sampler model). The probability of exploration at trial *t* is: $\varepsilon^{(t-1)/t}$.

In addition, SAW assumes that the estimation of the value of Option j under the exploitation mode is a weighted average of two estimates: Grand Mean ($GM_{j,t}$) – the option's mean payoff in the previous t -1 trials, and sample mean ($SM_{j,t}$) – the option's mean payoff in a sample of k_i past trials:

$$Evaluation_{j,t} = w_i(GM_{j,t}) + (1- w_t)\ SM_{j,t}$$

The weight of the grand mean w_i is 0.5 with probability ω (a free parameter), and 0 otherwise. The value of k_i is uniformly drawn from the set {1, 2…κ} where κ is a free parameter. The current predictions were derived with the parameters $\kappa = 9$, $\varepsilon = 0.4$, and $\omega = 0.5$. These values best fit the 88 conditions analyzed in Erev, Ert, Plonsky, and Roth (2020).

Appendix 2 – Experimental Instructions
First screen:

Welcome!

You are about to participate in an experiment on decision-making.
The experiment includes many rounds.
In each round, you will either win or lose some number of points.
Your final payoff at this experiment includes:

- an endowment of 1 $ plus
- $2 **for sure IF you made most of your choices on time** plus
- a chance to earn additional $1 bonus

IMPORTANTLY:

- This is a four person game, so please be considerate of the other participants, who cannot proceed without you!
- Please note usually you will have to **wait for a few minutes** until you are paired with a group.
- If you are not paired with a group, feel free to submit the assignment **AFTER 8 minutes**.
- The 8 minutes time will be STRICTLY enforced, if you submitted your assignment before 8 minutes passed, your assignment will be rejected.
- If you make most of the choices in the experiment on your own, you will receive additional $2 **for sure**.
- After you get paired, the experiment should proceeds smoothly.
- If you do not want to participate, please just close the browser NOW (without submitting), it will not harm your reputation in any way!

Please click "Next" if you agree to participate.

Next

Second Screen:

Welcome!

In this experiment, you are a part of a four participant group.
In each trial you and other three participants have to decide between strategies "A" and "B".
Your goal in this experiment is to maximize the accumulated number of points you earn.
The higher the number of points you earn, the higher your chance to earn additional $2 bonus.

The number of points you receive in each trial depends on your choice, the choices of others and luck.
Specifically:

- If you choose "A", and all other three participants choose "A" as well, you will get 0 points for sure.
- If you choose "A", and at least one other participant chooses "B", you will get 0 points with probability 0.98 and lose 60 points with probability 0.02.
- If you choose "B", you will win 1 point with probability 0.98 and lose 60 points with probability 0.02.

The game payoffs would remain on-screen for the entire experiment.

To clarify, "lose 0.2 points with probability 0.98 and lose 60 points with probability 0.02", means that roughly in 98 out of 100 times, the outcome will be "loses 0.2 points" and roughly 2 in 100 times, the outcome will be "loses 60 points".

In the first 3 rounds, you will have 20 seconds to make your choice and click on the "Next" button.
In each round after the first 3 rounds, you will have 12 seconds to make your choice and click on "Next" button.
If you do not make your choice and click "Next" by that time, the computer will make a choice for you, and you will lose 2 points (in addition to this round's payoff).

In the "Comments" text field on the next page, you have to enter the word "agree" (without the quotes), this is to ensure that you have read and understood the instructions and consent to participate.

Your final payoff at this experiment includes:

- an endowment of $1 plus
- $2 for **sure IF you made most of your choices on time** plus
- a chance to earn additional $1 bonus

Should you have any questions, please send an email to rothefim [at] gmail [dot] com.

Next
